# Digital PCR cluster predictor: a universal R-package and shiny app for the automated analysis of multiplex digital PCR data

**DOI:** 10.1093/bioinformatics/btad282

**Published:** 2023-04-22

**Authors:** Alfonso De Falco, Christophe M Olinger, Barbara Klink, Michel Mittelbronn, Daniel Stieber

**Affiliations:** National Center of Genetics (NCG), Laboratoire National de Santé (LNS), Dudelange 3555, Luxembourg; Faculty of Science, Technology and Medicine (FSTM), Luxembourg Center of Neuropathology (LCNP), University of Luxembourg (LNS), Belvaux 4367, Luxembourg; Department of Sports Medicine, Rehabilitation and Disease Prevention, University of Mainz, Mainz 55128, Germany; National Center of Genetics (NCG), Laboratoire National de Santé (LNS), Dudelange 3555, Luxembourg; National Center of Genetics (NCG), Laboratoire National de Santé (LNS), Dudelange 3555, Luxembourg; Department of Cancer Research (DoCR), Luxembourg Institute of Health (LIH), Luxembourg 1526, Luxembourg; Department of Cancer Research (DoCR), Luxembourg Institute of Health (LIH), Luxembourg 1526, Luxembourg; National Center of Pathology (NCP) and Luxembourg Center of Neuropathology (LCNP), Laboratoire National de Santé (LNS), Dudelange 3555, Luxembourg; Department of Life Science and Medicine (DLSM), Luxembourg Centre for Systems Biomedicine (LCSB), University of Luxembourg 4367, Belvaux, Luxembourg; National Center of Genetics (NCG), Laboratoire National de Santé (LNS), Dudelange 3555, Luxembourg

## Abstract

Digital polymerase chain reaction (dPCR) is an emerging technology that enables accurate and sensitive quantification of nucleic acids. Most available dPCR systems have two channel optics, with *ad hoc* software limited to the analysis of single and duplex assays. Although multiplexing strategies were developed, variable assay designs, dPCR systems, and the analysis of low DNA input data restricted the ability for a universal automated clustering approach. To overcome these issues, we developed dPCR Cluster Predictor (dPCP), an R package and a Shiny app for automated analysis of up to 4-plex dPCR data. dPCP can analyse and visualize data generated by multiple dPCR systems carrying out accurate and fast clustering not influenced by the amount and integrity of input of nucleic acids. With the companion Shiny app, the functionalities of dPCP can be accessed through a web browser.

## 1 Introduction

Digital polymerase chain reaction (dPCR), a third-generation PCR technology, relies on the distribution and individual amplification of target molecules in numerous partitions, thereby increasing amplification efficiency and overall sensitivity. Target molecules are detected using fluorescently labelled probes or intercalating dyes. Fluorescence values measured from each partition are reported in a 2D-color plot in which partitions containing the same targets co-localize within the same cluster. The number of positive partitions per target determines its amount and the concentration of a given target is then computed based on Poisson distribution. dPCR is well suited for applications requiring high sensitivity and precision such as rare allele detection, liquid biopsies, non-invasive prenatal testing, absolute quantification of viral load, single-cell gene expression, and genetically modified organism detection (Cao et al. 2017).

Most dPCR systems carry out the detection in two discrete optical channels limiting the multiplex capabilities of dPCR *ab initio*. Different approaches for the automated analysis of single and duplex assays (Trypsteen et al. 2015; Attali et al. 2016; Chiu et al. 2017) as well as for the classification of *rain* (Jones et al. 2014; Jacobs et al. 2017) were developed, with ‘*rain*’ referring to data elements localized between two clusters that cannot unambiguously be attributed to one of them. Several strategies for assay-multiplexing (amplitude-based, ratio-based, and non-discriminating multiplexing) (Whale et al. 2016) and the analysis of multiplex data were developed. ddPCRmulti is a semi-automated tool limited to the analysis of assays with orthogonal geometry (Dobnik et al. 2016), whereas ddPCRclust is an automated tool for the analysis of assays with non-orthogonal geometry (Brink et al. 2018). However, a universal automated tool for dPCR data analysis that is compatible with multiple- and diverse-multiplexing strategies and works well with limited amounts and quality of input nucleic acids is still missing.

To that end, we developed dPCR Cluster Predictor (dPCP), an R package and a companion Shiny app (Chang et al. 2020) for the automated analysis of multiplex dPCR data generated by orthogonal and non-orthogonal assays. dPCP is based on the combination of the density-based spatial clustering of applications with noise (DBSCAN) (Ester et al. 1996) and the c-means (Bezdek 1981) algorithms using a reference sample for the reliable classification of clusters with few elements. dPCP supports the analysis of data generated by both the Droplet Digital PCR System (Bio-Rad) and the QuantStudio 3D Digital PCR System (Applied Biosystems).

## 2 Methods

### 2.1 dPCR cluster predictor

**Figure 1.**
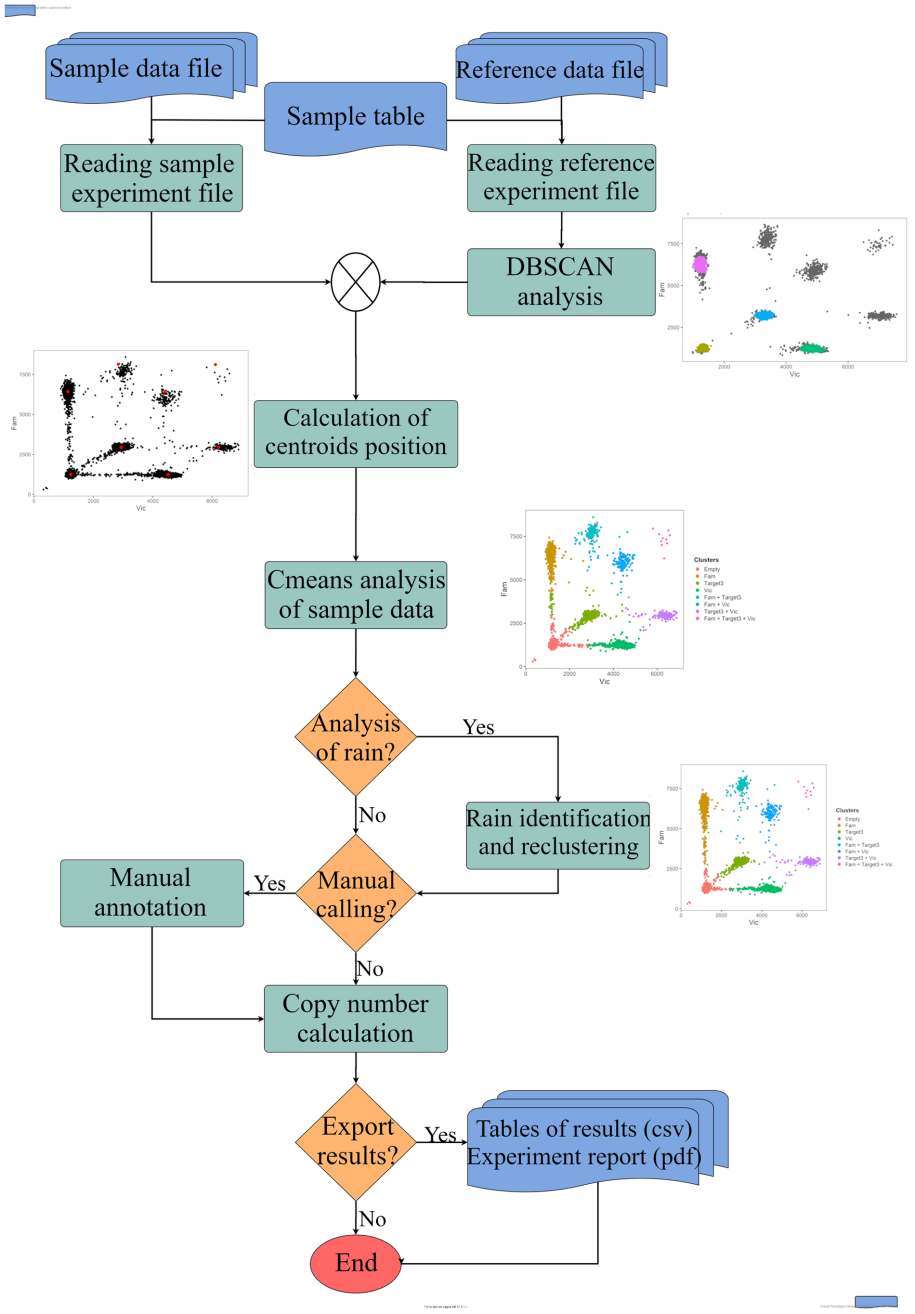
Flowchart of dPCP analysis. This flowchart illustrates the different analysis steps of dPCP during clustering analysis. The plots represent examples of intermediate analysis results.

dPCP uses two types of input files: a raw data file per sample and reference sample as well as a sample table (.csv file) containing information of experimental setup (Supplementary Tables S1 and S2). For each assay, a reference sample can be used as a scaffold for cluster analysis. The use of a reference sample is optional. However, dPCP requires a reference when the analysis of highly diluted samples is performed. A good reference is characterized by <5% of partitions tagged as rain and by the fact that all of its single-target clusters have been identified by the function *dbscan_combination* (Supplementary Fig. S1) available in the R package. During the analysis, the software carries out a quality control by assessing the number of single-target clusters and advises the user on the percentage of rain-tagged partitions in the reference.dPCP analyses data sequentially in seven steps (Fig. 1):

1. Reading experiment files and sample table.
2. Identification of empty partitions and single-target clusters in the reference using DBSCAN.
3. Calculating coordinates of clusters centroid. The distance between two cluster centroids can be represented by a Euclidean vector, therefore the coordinates of the centroids of multi-target clusters are predicted to be the sum of the coordinates of the centroids of single-target clusters. dPCP first calculates the arithmetic mean of the coordinates of all elements of clusters identified in step 2, identifying the centroid of single-target clusters, then predicts the position of the multi-target clusters by computing the vector sum (Supplementary Fig. S2).
4. Fuzzy c-means clustering. The centroid coordinates are used as input parameters for c-means clustering of the samples. The algorithm computes a membership matrix of probabilities of elements belonging to each cluster. Cluster membership is based on the highest rank.
5. Rain identification and re-clustering. Based on the membership matrix of the c-means algorithm a rain tag is assigned to all elements having as the highest probability a value ˂0.5. Rain-tagged elements are re-clustered by calculating the Mahalanobis distance (Mahalanobis 1936) to all the clusters and assigned to the cluster with the lowest value.
6. Optional: Manual correction. An interactive function has been implemented for manual correction of clustering after visual inspection of the data.
7. Copy number calculation. The target quantification is calculated based on Poisson distribution (Hindson et al. 2011). Precision is calculated as previously described (Majumdar et al. 2015).

All results and analysis information can be exported to a csv or pdf file. dPCP is available as R-package (https://cran.r-project.org/web/packages/dPCP/index.html) or web app (https://dpcp.lns.lu).

## 3 Results

To validate dPCP, it was compared to other methods available for the clustering of dPCR data (external validation). To that end, we used the adjusted Rand index (Rand 1971; Hubert and Arabie 1985) to measure the similarity of clustering obtained with dPCP and several reference methods. Furthermore, the quality of the dPCP clustering structure was evaluated using Silhouette analysis (Rousseeuw 1987) (Internal validation), and the results were interpreted according to established guidelines (Kaufman and Rousseeuw 1990) (Supplementary material and methods). First, dPCP analyses of singleplex, duplex, and higher-plex assays (dataset 1) were compared to QuantaSoft, QuantStudio 3D AnalysisSuite, and manual annotation, respectively. The samples analysed were purposefully heterogeneous in terms of type and integrity of template DNA, dPCR systems, number of targets, presence of rain, cluster separation, and multiplexing strategies (Supplementary Table S3). Additionally, dPCP was compared to ddPCRclust for the analysis of non-orthogonal multiplex assays (dataset 2). To that end, we used part of the dataset used for ddPCRclust validation (Brink et al. 2018) (Supplementary Table S4).

Based on the calculated adjusted Rand Index and on computed copy numbers, the results of dPCP clustering were highly comparable to all reference methods (adj. Rand Index range: 1–0.9884) (Supplementary Tables S3 and S4). Regarding cluster structure, the averages of Silhouette coefficients were ˃0.71 in all samples of dataset 1 (Supplementary Table S3) and dataset 2 (Supplementary Table S4), this confirms that dPCP identified clusters with a strong structure. Moreover, we show that dPCP is faster than ddPCRclust (2.83 s versus 16.91 s; *P*-value < .001) (Supplementary Table S4).

To estimate the sensitivity on diluted samples, a 10× 2-fold serial dilution (10–0.0195 ng) of genomic DNA was amplified with a triplex assay (Multiplex2). Data were analysed using dPCP and ddPCRclust, and results were compared to manual annotation that served as reference. Both the adjusted Rand index and the copy number calculation showed that dPCP was significantly more similar to manual annotation than ddPCRclust (*P*-value < 0.05), highlighting the difference between the two algorithms for low input amounts (Supplementary Table S5). The Silhouette coefficient of dPCP analysis for all samples was consistently high (0.9135–0.9471), demonstrating that dPCP is not influenced by the input amount whereas it systematically lowered for ddPCRclust an effect that increased with decreasing sample quantity (Supplementary Table S5).

## 4 Discussion

High sensitivity and accuracy for the detection and quantification of nucleic acids are defining hallmarks of dPCR enabling applications ranging from absolute quantification to rare allele detection. In this context, a universal and reliable automated algorithm for data analysis is crucial. However, the automated analysis of multiplex assays and clustering of data generated with limited input amount is missing. Here, we present dPCP a novel software for the analysis of dPCR data addressing those limitations. dPCP was compared to software developed by dPCR manufacturers, manual annotation, and ddPCRclust for the analysis of data generated from different sample types, using different assay designs, and dPCR systems.

Clustering results obtained with dPCP were consistent with results of all software we used for comparison. Many analytical applications rely on the capability of dPCR to detect rare events; therefore, an appropriate data analysis must be able to identify clusters with only a few data elements. To assess the performances of dPCP in this context, a DNA serial dilution was used as template for the detection of three target genes non-orthogonal assay and compared to manual annotation as reference method. The results showed that dPCP analysis retrieved comparable results to manual annotation and was superior to ddPCRclust, especially with highly diluted samples. Silhouette coefficient analysis of all the clustered datasets showed that dPCP achieves well-structured clustering even on highly diluted samples.

Analysis duration is an important performance indicator for algorithms. Manual analysis of big datasets is time-consuming, both ddPCRclust and dPCP are significantly faster, with dPCP being six times faster than ddPCRclust. A limitation of dPCP lies in the identification of multi-target clusters whose position significantly deviates from the calculated vector sum; this is common for competitive assays. However, the flexibility provided by the use of c-means algorithm allows to partly mitigate this limitation and to correctly identify multi-target clusters in some competitive assays. The robustness of the final clustering is assay dependent and is based on c-means performance. Moreover, the quality of the final clustering may be poor when the position of clusters and/or centroids in the sample data plot largely differs from those in the reference plot. This can be avoided by using the same experimental conditions between reference and unknown samples (e.g. using the same assay, primers and probe concentrations, and cycling protocol).

An index to evaluate the quality of the determination of centroids would be useful when there is a large deviation between the reference and a sample. This feature has not been implanted here, but the software allows for the visual inspection of this step of the pipeline. Additionally, the visual inspection allows to evaluate other features relevant to quality (e.g. cluster shape, separation of neighbouring clusters, and data density around the centroid) at the same time and to have a good overview of the software performance.

With this study, we demonstrate that dPCP is a flexible and reliable algorithm for the automated analysis of diverse dPCR data which is a unique feature in the landscape of methods currently available for dPCR data analysis. dPCP is available as R package or web app for non-R users.

## Supplementary Material

btad282_Supplementary_Data

## Data Availability

The data underlying this article are available at https://github.com/lns-lu/dpcp. R package: https://cran.r-project.org/web/packages/dPCP/index.html; https://github.com/alfodefalco/dPCP; Web: https://dpcp.lns.lu.
