# Supplementary material for: Digital PCR cluster predictor: a universal R-package and shiny app for the automated analysis of multiplex digital PCR data
Source: Bioinformatics. 2023 Apr 22;39(5):btad282. doi: 10.1093/bioinformatics/btad282 (PMC10168580; doi:10.1093/bioinformatics/btad282)
Supplement: btad282_Supplementary_Data [file btad282_supplementary_data.docx]

# **Supplementary material and methods**

## **Digital PCR Cluster Predictor**

The clustering process carried out by dPCP is mainly based on two algorithms:

1. Density-based spatial clustering of applications with noise (DBSCAN) algorithm (Ester *et al.*, 1996), a density-based non-parametric algorithm for data clustering that requires two input parameters: maximum distance (ε) between cluster elements and the number of minimum elements (minPts) to assemble a cluster. DBSCAN identifies a cluster when a number of data elements at least equal to the number of minimum elements are packed within the maximum distance ε. In the dPCR experiments, the empty partitions and single target clusters are easier to identify because their data density is higher than multi-target clusters; therefore, DBSCAN is used in the step 2 of dPCP workflow to identify the empty partitions and single-target clusters in the reference dataset.
2. In Fuzzy c-means algorithm (Bezdek, 1981), cluster analysis is based on minimizing the variance within the clusters. The intra-cluster variance is defined as the sum of the squared distance of all cluster elements from the cluster centroid.

The algorithm produces also a membership matrix in which the probability of elements of belonging to each cluster is represented. The probability is expressed with values between 0 and 1. The sum of all probabilities of a single element is 1.

For the development of dPCP the following R packages were used: *cluster*, *dbscan*, *e1071*, exactci, *ggplot2*, *ggpubr*, *graphics*, *raster*, *rlist*, *scales*, *shiny*, *shinyjs*, *stats*, *stringr*, *utils* (Maechler *et al.*, 2021; Hahsler *et al.*, 2019; Meyer *et al.*, 2021; Wickham, 2016; Kassambara, 2020; Hijmans, R. J. and van Etten, 2014; Ren, 2016; Wickham and Seidel, 2020; Chang *et al.*, 2020; Attali, 2020; Wickham, 2019; Fay, 2010).

## **Extraction and quantification of template DNA**

The following samples were used as the template for the dPCR experiments.

- human genomic DNA (Human Genomic DNA, Promega, Cat No: G1471),
- human cfDNA (5% Multiplex I cfDNA Reference Standard, Horizon Discovery, Cat. No: HD777),
- FFPE human DNA (Quantitative Multiplex Reference Standard, Horizon Discovery, Cat No: HD200)
- FFPE DNA was extracted from a single paraffin slide with QIAamp DNA FFPE Tissue Kit (Qiagen, Cat No: 56404) and Deparaffinization Solution (Qiagen, Cat No: 19093), adopting the following protocol: 320 µl of Deparaffinization Solution was added to the paraffin slide and incubated at 56 °C for 3 minutes, after the mixture was cooled at room temperature and 180 μl of Buffer ATL was added, the tube was centrifuged for 1 minute at 11,000 x g, 20 μl of proteinase K was added to the lower phase and mixed, the mixture was incubated at 56 °C overnight while shaking at 1000 rpm. The next day the lysate was incubated 1 minute at 90 °C, briefly centrifuged and the lower phase was transferred to a new tube to add and mix 200 μl of Buffer AL and 200 μl of ethanol (96–100%). After a brief centrifugation the lower phase is transferred to the QIAamp MinElute columns to perform a series of washing and the final elution in 100μl Buffer ATE.

DNA was quantified by fluorometry with the Qubit 2.0 (Invitrogen, Cat No: Q32866) and the dsDNA BR Assay kit (Invitrogen, Cat No: Q32850) or dsDNA HS Assay Kit (Invitrogen, Cat No: Q32851) following the manufacturer’s protocol.

## **Digital PCR**

dPCR runs were performed with QX200 Droplet Digital PCR System (Bio-Rad, Cat No: 1864001) and QuantStudio 3D Digital PCR System (Applied Biosystems, Cat No: 4489084) according to the manufacturer’s instructions.

QX200 Droplet Digital PCR System. The amplification mix (22 µl) was prepared with 11 µl of ddPCR Supermix for Probes (No dUTP) (Bio-Rad, Cat. No: 1863024), target primers and probes, 0.5 µl of restriction enzyme Hind III HF (New England BioLabs, Cat. No: R3104S) (used only in conjunction with high molecular weight DNA), and 5 µl of template DNA. 20 μl of the amplification mix and 70 µl of Droplet Generation Oil for Probes (Bio-Rad, Cat. No: 1863005) were pipetted into the appropriate wells of DG8™ Cartridges for QX200™/QX100™ Droplet Generator (Bio-Rad, Cat. No: 1864008). The cartridge was placed into a DG8 Cartridge Holder (Bio-Rad, Cat. No: 1863051), covered with DG8™ Gaskets for QX200™/QX100™ Droplet Generator (Bio-Rad, Cat. No: 1863009) and loaded into a QX200™ Droplet Generator (Bio-Rad, Cat. No: 1864002) to generate the droplets. 40 µl of emulsion mix were transferred into ddPCR™ 96-Well Plates (Bio-Rad, Cat. No: 12001925), which were successively placed into a Plate Support Block (Bio-Rad, Cat. No: 1814085) to be sealed with pierceable PCR Plate Heat Seal foil (Bio-Rad, Cat. No: 1814040) inside a PX1 PCR Plate Sealer (Bio-Rad, Cat. No: 1814000). The sealed plate was placed into a C1000 Touch™ Thermal Cycler with 96–Deep Well Reaction Module (Bio-Rad, Cat. No: 1851197) and DNA was amplified using the following protocol: 95 °C for 10 min, 40 cycles at 94 °C for 30 s then 60 °C for 30 s, followed by 98 °C for 10 min and final cooling to 4 °C (Devonshire *et al.*, 2014). After the amplification, the plate was placed into a QX200™ Droplet Reader (Bio-Rad, Cat. No: 1864003) for the droplet reading.

QuantStudio 3D Digital PCR System. The amplification mix (14.5 µl) was prepared with 7.25 µl of QuantStudio™ 3D Digital PCR Master Mix v2 (Applied Biosystems, Cat. No: A26358) variable amounts of target primers and probes, and 2 µl of template DNA. The loading of the amplification mix into a QuantStudio™ 3D Digital PCR 20K Chip (Applied Biosystems, Cat. No: A26316) was made using a QuantStudio™ 3D Digital PCR Chip Loader (Applied Biosystems, Cat. No: 4482592). Afterwards, the chip was placed into a QuantStudio™ 3D Digital PCR Chip Adapter Kit for Flat Block Thermal Cycler (Applied Biosystems, Cat. No: 4485513) to be loaded into a ProFlex™ 2 x flat PCR System (Applied Biosystems, Cat. No: 4484078).

The DNA was amplified with the following protocol for the assays Duplex 1 and Duplex 2: 96 °C for 10 min, 40 cycles at 60 °C for 2 min then 98 °C for 30 s, followed by 60 °C for 2 min and final cooling to 10 °C. The protocol for the amplification of the other assays was the following: 96 °C for 10 min, 40 cycles at 95 °C for 15 s then 60 °C for 1 min, followed by 60 °C for 2 min and final cooling to 10 °C. After the amplification, the chip was placed into a QuantStudio™ 3D Digital PCR Instrument (Applied Biosystems, Cat. No: 4489084) for the reading.

## **Statistical analysis**

The similarity of cluster analysis was evaluated with the adjusted Rand index which is a corrected-for-chance version of the Rand index (Rand, 1971; Hubert and Arabie, 1985) . The Rand index is calculated as the ratio of the number of concordant and total pairwise comparisons between two cluster analyses. The adjusted Rand index generates a random model to calculate the expected similarity of all pairwise comparisons and uses the resulting values to correct the Rand index. The adjusted Rand index values can vary between -1 and 1. When two cluster analyses are equal the adjusted Rand index is 1, negative values indicate that cluster concordance is less than expected from a random model.

Silhouette coefficient was used as a metric for the internal validation of cluster analysis (Rousseeuw, 1987). The coefficient calculation is based on cluster cohesion (similarity of data elements in the same cluster) and separation between different clusters. Cluster cohesion for a data element is measured as the average distance to the elements in the same cluster, whereas separation is calculated as the minimum average distance to points in different clusters. The Silhouette coefficient *s(i)* of a data element *i* is defined as follow:

$$s\left( i \right)=\frac{b\left( i \right)-a(i)}{max\{a\left( i \right), b\left( i \right)\}}$$

where *a(i)* is the mean distance between *i* and all other data elements in the same cluster and *b(i)* is the mean of the distance from *i* to all elements of other clusters. In this study the Euclidean distance was used to calculate the distance between two data elements. The arithmetic mean of the Silhouette coefficients of all data elements is calculated and used to evaluate the quality of the clustering results. The interpretation of Silhouette coefficient given by Kaufman and Rousseeuw was used to interpret the results of Silhouette analyses (Kaufman and Rousseeuw, 1990). According to this interpretation, the mean value of Silhouette coefficient of all data elements in a sample gives an indication about the clustering architecture organized by an algorithm:

- mean value between 0.71 and 1: a strong structure has been found;
- mean value between 0.51 and 0.70: a reasonable structure has been found;
- mean value between 0.26 and 0.50: the structure is weak and could be artificial;
- mean value lower than 0.25: no substantial structure has been found.

The Pearson chi-square test was used as normality test to evaluate the data distribution of the time of analysis and adjusted Rand index. The time of analysis dataset was normally distributed and was thus described with mean and standard error of the mean (SEM), whereas median and interquartile range (IQR) were used to describe the non-normally distributed data of the adjusted Rand index. The difference of two groups of data was assessed using the paired t-test for normally distributed and the paired Wilcoxon test for non-normally distributed data respectively.

The statistical analysis and calculations of this study were computed with R using the following packages: *nortest* (Gross and Ligges, 2015) for the normality tests; *stats* for the t-test, Wilcoxon test, and the calculation of median and interquartile range; *mcclsust* (Fritsch, 2022) for the calculation of adjusted Rand index; *cluster* (Maechler *et al.*, 2021) to calculate the Silhouette coefficient and *ggplot2* (Wickham, 2016) for the graphics.

# **Supplementary figures**


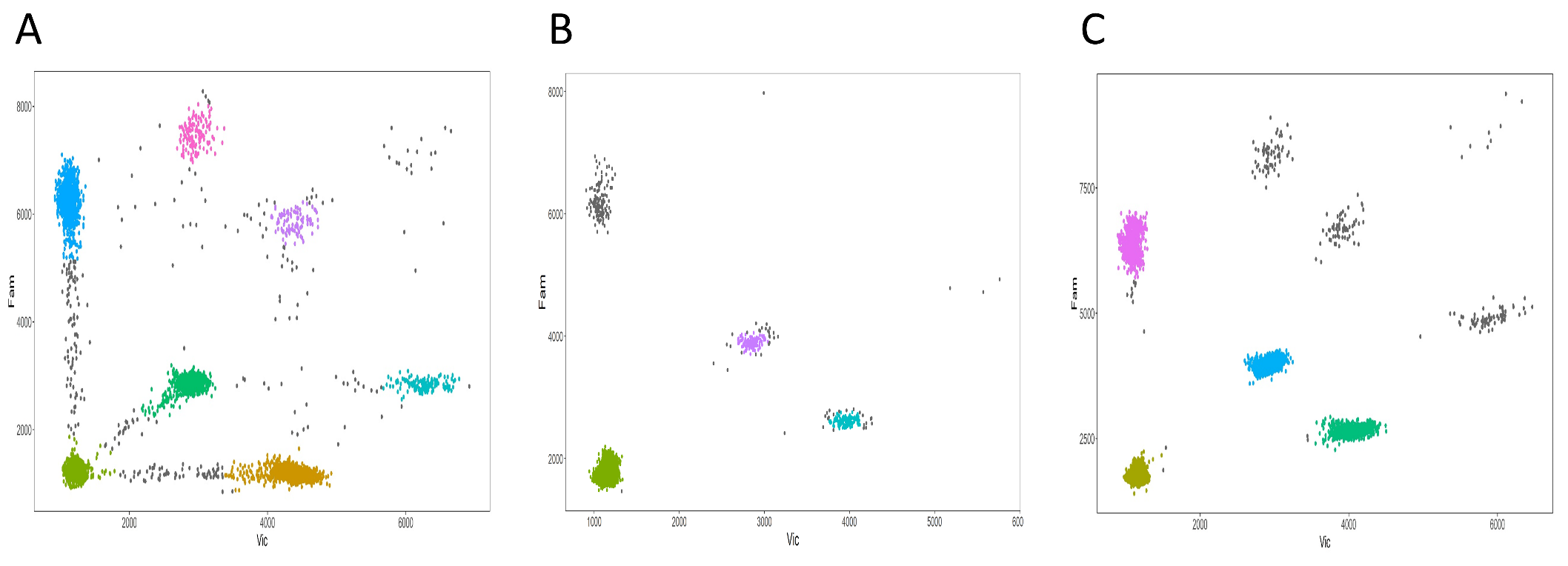


**Supplementary Figure 1. Evaluation of putative reference samples.** Each graph shows a DBSCAN analysis of a possible reference. Assembled clusters are represented with colored dots; different colors indicate distinct clusters and grey dots represent not-clustered elements.

In sample A, all single-target clusters and the empty partition cluster have been identified, however the poor quality of input nucleic acid results in important formation of rain.

The sample B does not show rain but one of the single-target cluster is not identified.

The sample C presents all the features necessary for a reference: absence of rain and identification of all single-target clusters and the empty partition cluster.


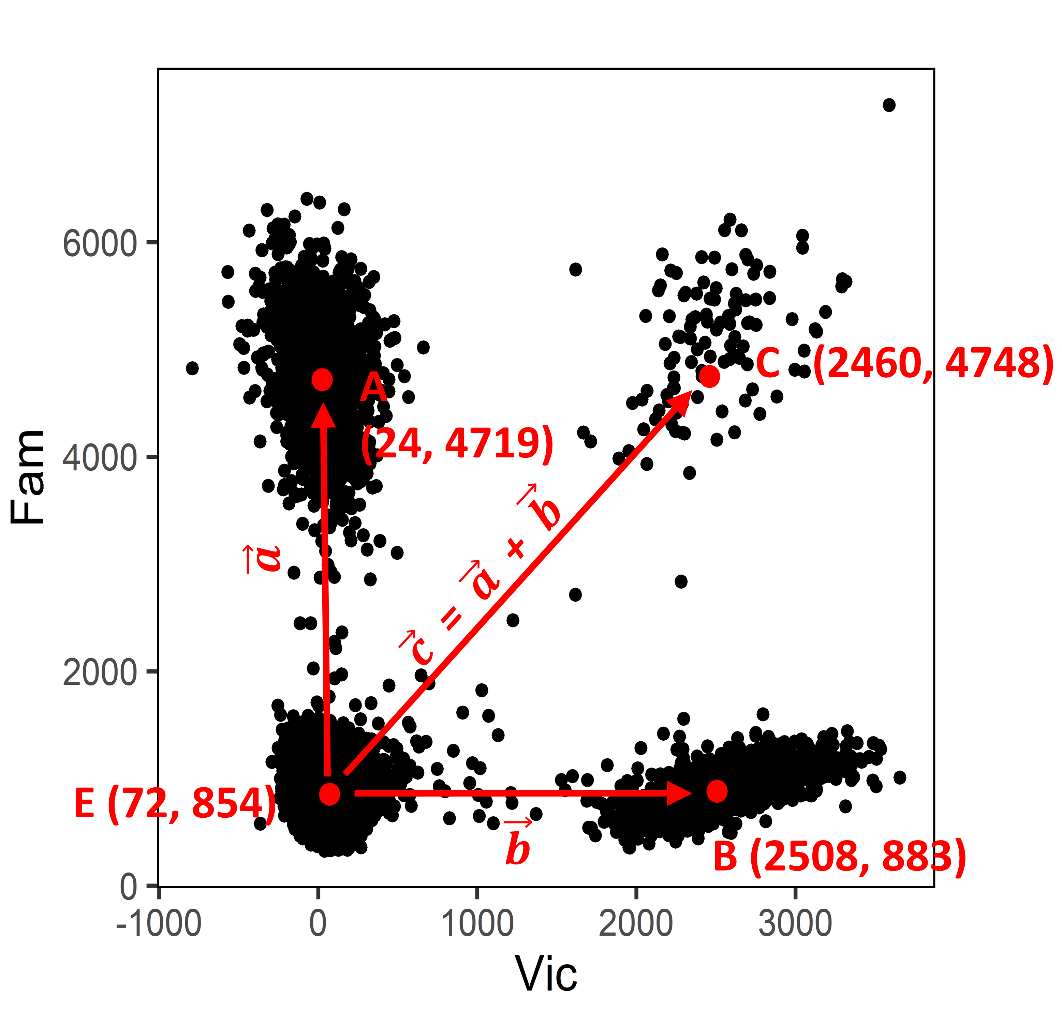


**Supplementary Figure 2.** **Identification of multi-target cluster centroids.** Fluorescence values of a duplex assay are plotted; red dots and values in brackets indicate the cluster centroid position and coordinates.

The coordinates of centroids E, A, and B were calculated by computing the arithmetic mean of fluorescence values of elements identified in step 2 of the dPCP pipeline. Coordinates of centroids E and A can be considered as the Cartesian representation of Euclidean vector $\vec{a}$, whereas the coordinates of centroid E and B can be considered as the Cartesian representation of Euclidean vector $\vec{b}$. The position of the centroid of the multi-target cluster was predicted from the coordinates of the centroid of single target clusters: dPCP calculated the coordinates of centroid C by computing the vector sum$\vec{a}+ \vec{b}$.

# **Supplementary tables**

**Supplementary Table 1. Example of the sample table for the analysis of data generated with the QX100/QX200 Droplet Digital PCR System.**

Chip ID/ Well ID, No of targets and Dilution are mandatory fields, all other information can be omitted.

| Sample name | Chip ID/ Well ID | No of targets | FAM target | Target 3 | Target 4 | VIC/HEX target | Reference | Dilution |
| --- | --- | --- | --- | --- | --- | --- | --- | --- |
| Sample1 | A01 | 3 | TBP |  | NAGK | RPPH1 |  | 1 |
| Sample2 | B01 | 2 | HBB |  |  | CYP2 | S2_reference_eps200_minPts50_DB.rds | 0.5 |
| Sample3 | C01 | 4 | 18S | GAPDH | ACTB | CYP1 | ref_01.csv | 0.2 |
| Sample4 | D01 | 2 | EGFR L858R |  |  | EGFR WT | ref20200312_A01_Amplitude.csv | 1 |
|  | E01 | 3 |  |  |  |  |  | 1 |

**Supplementary Table 2. Example of the sample table for the analysis of data generated with the QuantStudio 3D Digital PCR System.**

Chip ID/ Well ID, No of targets and Dilution are mandatory fields, all other information can be omitted.

| Sample name | Chip ID/ Well ID | No of targets | FAM target | Target 3 | Target 4 | VIC/HEX target | Reference | Dilution |
| --- | --- | --- | --- | --- | --- | --- | --- | --- |
| Sample1 | D01G97 | 3 | TBP |  | NAGK | RPPH1 |  | 1 |
| Sample2 | D01HRW | 2 | HBB |  |  | CYP2 | C06BOO_qlty0.5_eps200_minPts50_DB.rds | 0.5 |
| Sample3 | D01HSE | 4 | 18S | GAPDH | ACTB | CYP1 | C0HYMM | 0.2 |
| Sample4 | D01HQR | 2 | EGFR L858R |  |  | EGFR WT | C0HYMM | 1 |
|  | D01HGH | 3 |  |  |  |  |  | 1 |

**Supplementary Table 3. Dataset 1.** dPCP comparison to QuantaSoft, QuantStudio 3D Analysis Suite software and manual annotation.

*Assays details are reported in the Supplementary Table 6 and 7, and Supplementary material and methods.

|  | | | External clustering validation | | | Internal clustering validation | | Copies/µl | | |
| --- | --- | --- | --- | --- | --- | --- | --- | --- | --- | --- |
| **Assay*** | **Template DNA*** | **dPCR System** | **Reference method** | **Adjusted Rand index** | **Silhouette coefficient (mean)** | | **Reference method** | | **dPCP** |  |
| Singleplex 1 | Reference genomic | QX200 | QuantaSoft | 0.9994 | 0.9695 | | 84.5 | | 84.4 |  |
| Singleplex 2 | Reference genomic | QX200 | QuantaSoft | 0.9994 | 0.9785 | | 88.6 | | 88.8 |  |
| Singleplex 3 | Reference genomic | QX200 | QuantaSoft | 1 | 0.9808 | | 98.7 | | 98.0 |  |
| Duplex 1 | Reference FFPE | Quant Studio | Quant Studio 3D Analysis Suite | 0.9990 | 0.8725 | | 844.8  29.5 | | 846.5  30.6 |  |
| Duplex 2 | Reference cfDNA | Quant Studio | Quant Studio 3D Analysis Suite | 0.9995 | 0.9112 | | 357.8  17.5 | | 358.8  18.0 |  |
| Duplex 3 | Reference genomic | Quant Studio | Quant Studio 3D Analysis Suite | 0.9999 | 0.8661 | | 112.5  119.6 | | 112.5  119.5 |  |
| Orthogonal  Multiplex 1 | Reference genomic | Quant Studio | Manual annotation | 0.9946 | 0.7882 | | 202.6  206.7  207.1 | | 202.5  225.6  208.2 |  |
| Non-orthogonal  Multiplex 2 | Reference FFPE | QX200 | Manual annotation | 0.9966 | 0.9250 | | 131.2  123.7  119.3 | | 132.1  123.5  120.0 |  |
| Non-orthogonal  Multiplex 3 (Brink *et al.*, 2018) | Blood DNA | QX200 | Manual annotation | 0.9992 | 0.9244 | | 233.1  228.2  229.3  235.2 | | 232.7  226.5  229.7  234.4 |  |

**Supplementary Table 4. Dataset 2.** dPCP comparison to ddPCRclust.

| Sample  (DNA source) | Number  of targets | Adjusted Rand index | dPCP Silhouette coefficient (mean) | ddPCRclust  time of analysis (s) | dPCP  time of analysis (s) | Copies/µl | |
| --- | --- | --- | --- | --- | --- | --- | --- |
|  |  |  |  |  |  | **ddPCRclust** | **dPCP** |
| G08 (Cell line) | 3 | 0.9992 | 0.9549 | 8.95 | 1.11 | 350.3  387.3  373.1 | 350.2  386.4  373.2 |
| B01 (Blood) | 4 | 0.9965 | 0.9244 | 10.72 | 1.18 | 233.1  228.2  229.3  235.2 | 232.7  226.5  229.7  234.4 |
| F02 (Blood) | 3 | 0.9995 | 0.9749 | 25.94 | 4.22 | 77.3  75.6  76.6 | 77.3  75.7  76.5 |
| G01 (FFPE) | 4 | 0.9884 | 0.8805 | 8.79 | 1.81 | 131.0  136.2  141.2  133.9 | 123.7  141.7  139.7  132.3 |
| E09 (FFPE) | 2 | 0.9906 | 0.9643 | 21.33 | 3.62 | 95.3  117.4 | 93.3  116.6 |
| A04 (FFPE) | 4 | 0.9917 | 0.9477 | 20.27 | 3.93 | 42.4  41.3  50.9  45.3 | 38.3  45.1  49.8  44.7 |
| D03 (FFPE) | 3 | 0.9891 | 0.9575 | 22.39 | 3.95 | 28.8  34.9  43.7 | 28.4  33.8  43.1 |
| Mean (SEM) |  |  |  | 16.91 (3.85) | 2.83 (0.75) |  |  |

**Supplementary Table 5. Cluster analysis of DNA serial dilution.**

*In low input samples, ddPCRclust did not identify any cluster other than the empty partitions, impeding the calculation of the Silhouette coefficient.

|  | Adjusted Rand index | | Silhouette coefficient (mean) | | Copies/µl – 3 targets | | | | |
| --- | --- | --- | --- | --- | --- | --- | --- | --- | --- |
| **Sample** | **dPCP –**  **manual annotation** | **ddPCRclust –**  **manual annotation** | **dPCP** | **ddPCRclust*** | **Manual annotation** | **dPCP** | **ddPCRclust** | | |
| 10  ng | 0.9995 | 0.9969 | 0.9135 | 0.9094 | 180.2  173.7  174.4 | 180.3  173.0  175.0 | 180.6  174.5  176.0 | | |
| 5  ng | 0.9989 | 0.9957 | 0.9273 | 0.9245 | 87.6  93.4  90.3 | 87.6  93.0  91.0 | 87.6  94.2  91.8 | | |
| 2.5  ng | 0.999 | 0.9968 | 0.9403 | 0.9334 | 38.9  41.5  39.2 | 38.9  41.7  39.2 | 39.0  43.3  40.9 | | |
| 1.25  ng | 0.9992 | 0.8482 | 0.9405 | 0.4538 | 19.0  20.5  19.8 | 19.0  20.5  19.9 | 39.6  37.0  55.5 | | |
| 0.625 ng | 1 | 0.9994 | 0.9446 | 0.8930 | 9.8  8.9  10.2 | 9.8  8.9  10.2 | 399.76  4387.3  10.0 | | |
| 0.31  ng | 1 | 0.2739 | 0.9471 | 0.3363 | 5.1  3.7  4.3 | 5.1  3.7  4.3 | 5.6  71.5  7.9 | | |
| 0.156 ng | 1 | 0.0457 | 0.939 | - | 3.3  2.3  2.3 | 3.3  2.3  2.3 | 0  0  0 | | |
| 0.078 ng | 1 | 0.0469 | 0.9455 | - | 1.5  0.83  1.5 | 1.5  0.83  1.5 | 0  0  0 | | |
| 0.039 ng | 1 | 0.9119 | 0.9436 | - | 0.81  0.67  0.61 | 0.81  0.67  0.61 | 0  0  0 | | |
| 0.0195 ng | 1 | 0.8748 | 0.9440 | - | 0.16  0.24  0.16 | 0.16  0.24  0.16 | 0  0  0 | | |
| Median (IQR) | 1 (0.0007) | 0.8933 (0.5791) |  |  |  |  | |  |  |

**Supplementary Table 6. Primers and probes information.** Primers and probes sequences were taken from the cited publications or vendor or designed with Primer3Plus and their specificity was assessed with Primer-BLAST.

| **Gene symbol (accession number)** | **Primer/probe sequence (5’-3’)** | **Concentration Final (µM)** | **Amplicon length (bp)** |
| --- | --- | --- | --- |
| NAGK  (NC_000002.12) | Fw: TGGGCAGACACATCGTAGCA  (Devonshire *et al.*, 2014) | 0.2 | 66 |
|  | Rv: CACCTTCACTCCCACCTCAAC  (Devonshire *et al.*, 2014) |  |  |
|  | P: FAM-TGTTGCCCGAGATTGACCCGGT-BHQ1  (Devonshire *et al.*, 2014) | 0.1 |  |
|  | P: YAK-TGTTGCCCGAGATTGACCCGGT-BHQ1 | 0.15 |  |
| TBP  (NC_000006.12) | Fw: CACCTTACGCTCAGGGCTTG | 0.4 | 68 |
|  | Rv: TCCGCCCTCCCTATTCTCTC |  |  |
|  | P: FAM- CTCCCCT+CAGGTAATAT+AGCAGG-BHQ1 | 0.2 |  |
| HBB  (NC_000011.10) | Fw: GGGCAACGTGCTGGTCTG  (Klaassen *et al.*, 2003) | 0.25 | 71 |
|  | Rv: AGGCAGCCTGCACTGGT  (Klaassen *et al.*, 2003) |  |  |
|  | P: FAM-CTGGCCCATCACTTTGGCAAAGAA-BHQ1  (Klaassen *et al.*, 2003) | 0.1 |  |
| EGFR  (NC_000007.14) | EGFR_6224 Assay (Applied Biosystems, Assay ID: HS000000026_rm, Cat No: A44177) | - | - |
| RPPH1  (NC_00014.9) | Fw: GCGGAGGGAAGCTCATCAG  (Devonshire *et al.*, 2014) | 0.7 | 64 |
|  | Rv: GGACATGGGAGTGGAGTGACA  (Devonshire *et al.*, 2014) |  |  |
|  | P: YAK--CACG+AGCT+GAG+TGCG--BHQ1 | 0.25 |  |

**Supplementary Table 7. Digital PCR assays details.**

| **Assay** | **Number of targets** | **Target genes** |
| --- | --- | --- |
| Singleplex 1 | 1 | NAGK |
| Singleplex 2 | 1 | TBP |
| Singleplex 3 | 1 | RPPH1 |
| Duplex 1 | 2 | EGFR WT  EGFR L858R |
| Duplex 2 | 2 | EGFR WT  EGFR L858R |
| Duplex 3 | 2 | RPPH1  TBP |
| Multiplex 1 | 3 | HBB  NAGK  RPPH1 |
| Multiplex 2 | 3 | NAGK  RPPH1  TBP |
| Multiplex 3 | 4 | Sample B01, (Brink et al., 2018) |

**References**

Attali,D. (2020) shinyjs: Easily Improve the User Experience of Your Shiny Apps in Seconds.

Bezdek,J.C. (1981) Pattern Recognition with Fuzzy Objective Function Algorithms Springer US, Boston, MA.

Brink,B.G. *et al.* (2018) DdPCRclust: An R package and Shiny app for automated analysis of multiplexed ddPCR data. *Bioinformatics*, **34**, 2687–2689.

Chang,W. *et al.* (2020) shiny: Web Application Framework for R.

Devonshire,A.S. *et al.* (2014) Towards standardisation of cell-free DNA measurement in plasma: Controls for extraction efficiency, fragment size bias and quantification. *Anal. Bioanal. Chem.*, **406**, 6499–6512.

Ester,M. *et al.* (1996) A Density-Based Algorithm for Discovering Clusters in Large Spatial Databases with Noise. In, *Proceedings of the 2nd International Conference on Knowledge Discovery and Data Mining*., pp. 226–231.

Fay,M.P. (2010) Two-sided Exact Tests and Matching Confidence Intervals for Discrete Data. *R J.*, **2**, 53–58.

Fritsch,A. (2022) mcclust: Process an MCMC Sample of Clusterings.

Gross,J. and Ligges,U. (2015) nortest: Tests for Normality.

Hahsler,M. *et al.* (2019) dbscan: Fast Density-Based Clustering with R. *J. Stat. Softw.*, **91**, 1–30.

Hijmans, R. J. and van Etten,J. (2014) raster: Geographic data analysis and modeling. R package.

Hubert,L. and Arabie,P. (1985) Comparing partitions. *J. Classif.*, **2**, 193–218.

Kassambara,A. (2020) ggpubr: ‘ggplot2’ Based Publication Ready Plots.

Kaufman,L. and Rousseeuw,P.J. (1990) Finding Groups in Data: An Introduction to Cluster Analysis.

Klaassen,C.H.W. *et al.* (2003) Quantification of human DNA in feces as a diagnostic test for the presence of colorectal cancer. *Clin. Chem.*, **49**, 1185–1187.

Maechler,M. *et al.* (2021) cluster: Cluster Analysis Basics and Extensions.

Meyer,D. *et al.* (2021) e1071: Misc Functions of the Department of Statistics, Probability Theory Group (Formerly: E1071), TU Wien.

Rand,W.M. (1971) Objective Criteria for the Evaluation of Clustering Methods. *J. Am. Stat. Assoc.*, **66**, 846.

Ren,K. (2016) rlist: A Toolbox for Non-Tabular Data Manipulation.

Rousseeuw,P.J. (1987) Silhouettes: A graphical aid to the interpretation and validation of cluster analysis. *J. Comput. Appl. Math.*, **20**, 53–65.

Wickham,H. (2016) ggplot2: Elegant Graphics for Data Analysis Springer-Verlag New York.

Wickham,H. (2019) stringr: Simple, Consistent Wrappers for Common String Operations.

Wickham,H. and Seidel,D. (2020) scales: Scale Functions for Visualization.
